# Consciousness alteration in focal epilepsy is related to loss of signal complexity and information processing

**DOI:** 10.1038/s41598-022-25861-4

**Published:** 2022-12-24

**Authors:** Nada El Youssef, Aude Jegou, Julia Makhalova, Lionel Naccache, Christian Bénar, Fabrice Bartolomei

**Affiliations:** 1 APHM, Timone Hospital, Epileptology and Cerebral Rhythmology, Marseille, France; 2 Aix Marseille Univ, INSERM, INS, Inst Neurosci Syst, Marseille, France; 3 APHM, Timone Hospital, CEMEREM, Marseille, France; 4 APHP, Departments of Neurology & Clinical Neurophysiology Pitié Salpêtrière Hospital, Paris, France; 5 Service d’Epileptologie et de Rythmologie Cérébrale, Hôpital Timone, 264 Rue Saint-Pierre, 13005 Marseille, France

**Keywords:** Epilepsy, Neuroscience, Consciousness

## Abstract

Alteration of awareness is a main feature of focal epileptic seizures. In this work, we studied how the information contained in EEG signals was modified during temporal lobe seizures with altered awareness by using permutation entropy (PE) as a measure of the complexity of the signal. PE estimation was performed in thirty-six seizures of sixteen patients with temporal lobe epilepsy who underwent SEEG recordings. We tested whether altered awareness (based on the Consciousness Seizure Score) was correlated with a loss of signal complexity. We estimated global changes in PE as well as regional changes to gain insight into the mechanisms associated with awareness impairment. Our results reveal a positive correlation between the decrease of entropy and the consciousness score as well as the existence of a threshold on entropy that could discriminate seizures with no alteration of awareness from seizures with profound alteration of awareness. The loss of signal complexity was diffuse, extending bilaterally and to the associative cortices, in patients with profound alteration of awareness and limited to the temporal mesial structures in patients with no alteration of awareness. Thus PE is a promising tool to discriminate between the different subgroups of awareness alteration in TLE.

## Introduction

Temporal lobe epilepsy (TLE) is the most common form of focal epilepsy. Amongst the clinical manifestations of TLE seizures, alteration of consciousness is one of the most dramatic features, affecting 60–80% of patients^1^ and impacting quality of life. This justifies research efforts towards a better understanding of the mechanisms of loss of consciousness and towards finding more effective therapies^2^. Consciousness is a complex concept, which depends on the interaction between bilateral cortical networks and subcortical components, forming the “consciousness system”^3,4^. Alteration of consciousness during focal seizures can be conceptualized in a two-dimensional view of consciousness^2^ by integrating two main components: awareness, subserved by the cortico-cortical networks^5^, and wakefulness, associated with reticulo-thalamo-cortical projections^6^. However, previous work in focal epilepsy has mainly focused on alteration of the level of awareness. In the new ILAE classification of seizure types, “consciousness” was thus replaced by “awareness “^7^, which refers to the awareness of events occurring during a seizure^8^.

Two main theories have been proposed to account for the alteration of awareness during focal seizures: the “global workspace theory” (GWT)^2,9,10^ and “the network inhibition hypothesis”^3,4,11,12^. The GWT suggests that coordinated activity from the associative cortices (mainly the prefrontal cortex and the posterior parietal associative cortex) is needed to access consciousness from different sensory modalities^13^. Alteration of awareness in TLE could be linked to the alteration of the global workspace through hypersynchronization between cortical and subcortical structures (thalamo-cortical connections)^14^. The degree of thalamo-cortical synchrony has been shown to correlate with the degree of consciousness changes^14–16^ and consciousness changes have been related to hypersynchrony in the beta/gamma range^10,17^. The “network inhibition hypothesis”^3,4,11,12^ postulates the deactivation of cortical regions through inhibition of subcortical arousal systems affected by ictal discharge, resulting in neocortical inhibition and emergence of slow delta rhythms at the level of the frontoparietal neocortex.

In parallel, efforts have been made to quantify clinical and electrophysiological phenomena associated with altered consciousness in focal seizures. Three psychometric scales have been specifically proposed to measure ictal consciousness/awareness changes: the Ictal Consciousness Inventory (ICI)^18^, the Consciousness Seizure Scale (CSS)^14^ and the Responsiveness in Epilepsy Scale—versions I and II (RES-I and RES-II)^19^. The CSS is based on eight criteria measured by an epileptologist, with good inter and intra-observer reliability^14^.

Many neurophysiological methods have been used to quantify the complexity of signals across consciousness states, essentially in the domain of disorders of consciousness (DoC) such as coma or vegetative states. Entropy has been widely applied for quantification of brain activity, from the evaluation of consciousness alteration to assessment of brain networks interactions and the “ageing brain”^20^. Permutation entropy (PE) as proposed by Bandt^21^, is a method to measure the complexity of dynamic systems. It thus allows assessment of dynamic changes in EEG recordings, with higher entropy being associated with the higher complexity of signals.

In this study based on intracerebral stereotactic-EEG recordings (SEEG), we computed delta entropy, (∆E), corresponding to the decrease from baseline, and delta time metrics, derived from the permutation entropy, and correlated this measure to the awareness changes occurring during temporal lobe seizures. We tested whether altered awareness was correlated with the loss in signal complexity, a hypothesis previously formulated from connectivity data^9^. Finally, we estimated global as well as regional changes in PE to gain insight into the mechanisms associated with awareness impairment.

## Results

Sixteen patients with TLE were selected based on a clinical examination during the seizure that was informative enough to obtain a Consciousness Seizure Scale (CSS) score (Table 1). The CSS^14^ takes into account different components of consciousness leading to a composite score from 0 (no alteration of consciousness) to 9 (complete alteration of consciousness) and allows to distinguish different groups according to the extent of alteration of consciousness, with group A (score 0–1) corresponding to preserved consciousness, group B (score 2–5) to moderate alteration of consciousness and group C (score 6–9) to profound alteration of consciousness.

**Table 1:** Patients characteristics.

Patient number	Age at time of SEEG (Y)	Gender	Epilepsy duration	Etiology	Epilepsy subtype	Side	Surgery	Engel	Number of analyzed seizure/LOC	Number of Seizure according to CSS (A/B/C)
1	28	F	9	HS	MTLE	L	No	N/A	4/2	2/1/1
2	21	F	13	DNET	LTLE	R	Yes	4	3/0	3/0/0
3	36	F	21	HH	LTLE	Bil	No	N/A	1/0	1/0/0
4	30	F	9	PVHT	LTLE	R > L	No	N/A	2/1	1/1/0
5	27	F	10	HS	MTLE	R	Yes	1a	2/0	2/0/0
6	26	F	3	GN	MTLE	R	Yes	1a	2/2	0/0/2
7	26	M	26	PS	LTLE	R > L	No	N/A	2/2	0/1/1
8	44	M	4	TBI	LTLE	R	Yes	1a	3/3	0/3/0
9	45	F	18	HS	MTLE	L	Yes	1a	5/5	0/1/4
10	35	F	19	HS	MTLE	L	Yes	2a	1/1	0/0/1
11	33	M	22	TBI	LTLE	Bil	No	N/A	5/5	0/0/5
12	56	M	45	FCD	LTLE	R	Yes	1a	2/2	0/0/2
13	22	F	14	TBI	LTLE	R	Yes	3	1/1	0/0/1
14	44	M	15	UNK	LTLE	L	No	N/A	1/1	0/0/1
15	46	F	8	UNK	LTLE	L	Yes	3	1/1	0/0/1
16	29	F	10	UNK	LTLE	L	Yes	N/A	1/1	0/0/1

HS: hippocampal sclerosis; DNET: dysembryoplastic neuroepithelial tumor; GN: ganglioglioma; PNH: periventricular nodular heterotopia; HH: hypothalamic hamartoma; PS: perinatal stroke; TBI: traumatic brain injury; FCD: focal cortical dysplasia; Bil: bilateral; Unk: unknown.

We excluded secondary generalized seizures and seizures that were not tested within the first 30 s after electrical onset. A total of thirty-six seizures from sixteen subjects were analyzed. According to the CSS scores, nine seizures were classified into group A, seven seizures into group B, and 20 seizures into group C, respectively.

### Comparison of permutation entropy between the three seizure groups

**Figure 1:**
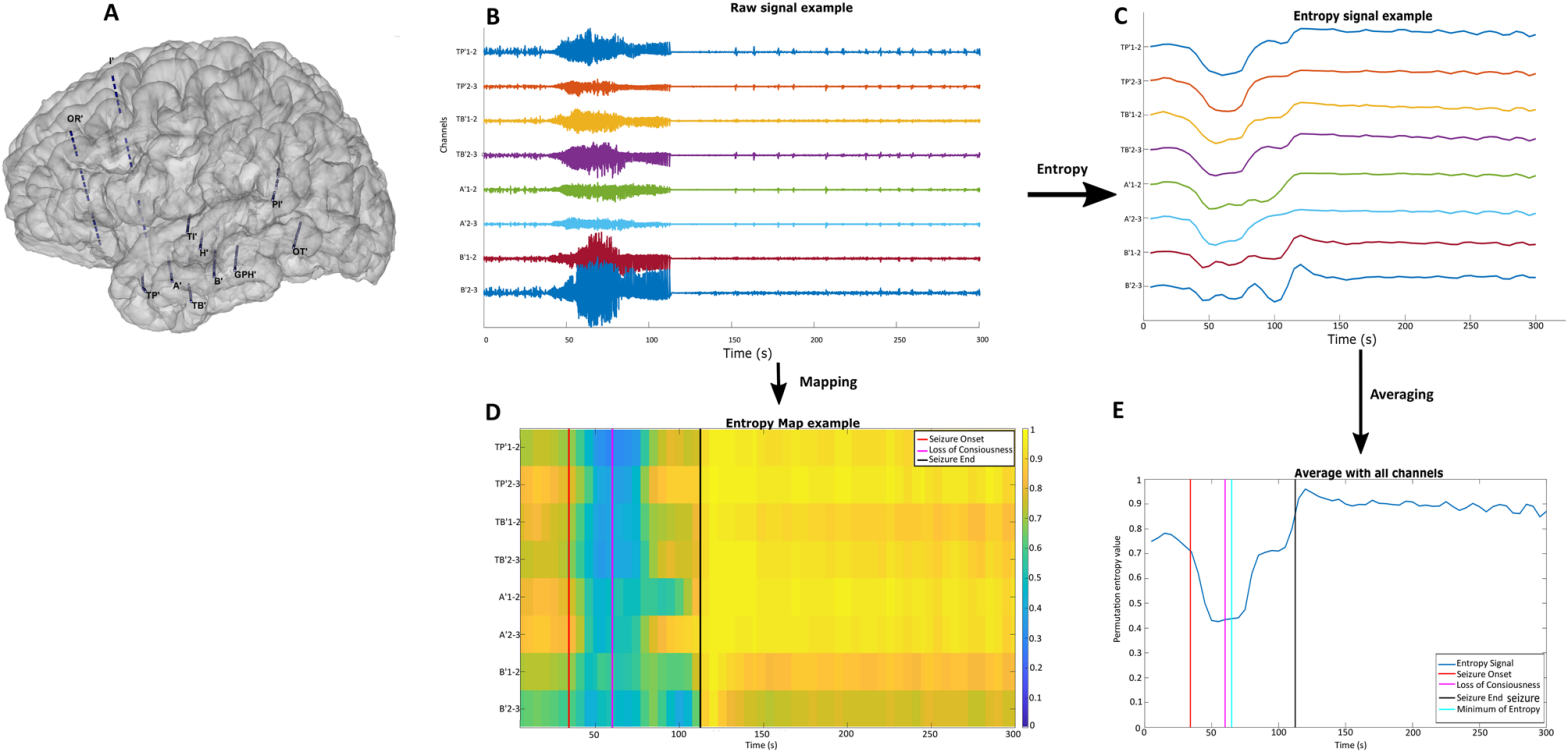
Illustration of the Permutation Entropy estimation. (**A**) 3D Representation of the stereotactic-EEG electrodes in one patient. (**B**) Electrophysiological signal associated to 8 different selected contacts of the implanted channels (bipolar montage). (**C**) Permutation entropy signal of those channels showing the decrease of values after seizure onset. (**D**) Map Representation of the entropy signal with the different markers, as set by the scorer, corresponding to the beginning of the seizure (red), the maximal loss of consciousness (pink) and the end of the seizure (black). (**E**) Average of the entropy signal of all the channels during a seizure with the following markers: Beginning of the seizure (red), Loss of consciousness (pink), minimum of entropy value (cyan) (this marker is used to determine the delta entropy) and the end of seizure (black). The red and black markers are determined by the scorer based on the electrophysiological signal; the pink marker is determined by the scorer based on the CSS; the cyan marker is determined in an automated way. Arrow means “after applying”.

Permutation entropy (PE) was computed on each channel, on a sliding window. The PE time courses of all seizures within each group were superimposed after subtraction of the mean entropy value of the baseline from the entropy value of the signal. Figure 1 illustrates changes in PE for 8 selected channels in one patient. “Delta entropy” (ΔE) is the difference between the entropy value at the baseline and the minimum entropy reached during the seizure.

**Figure 2:**
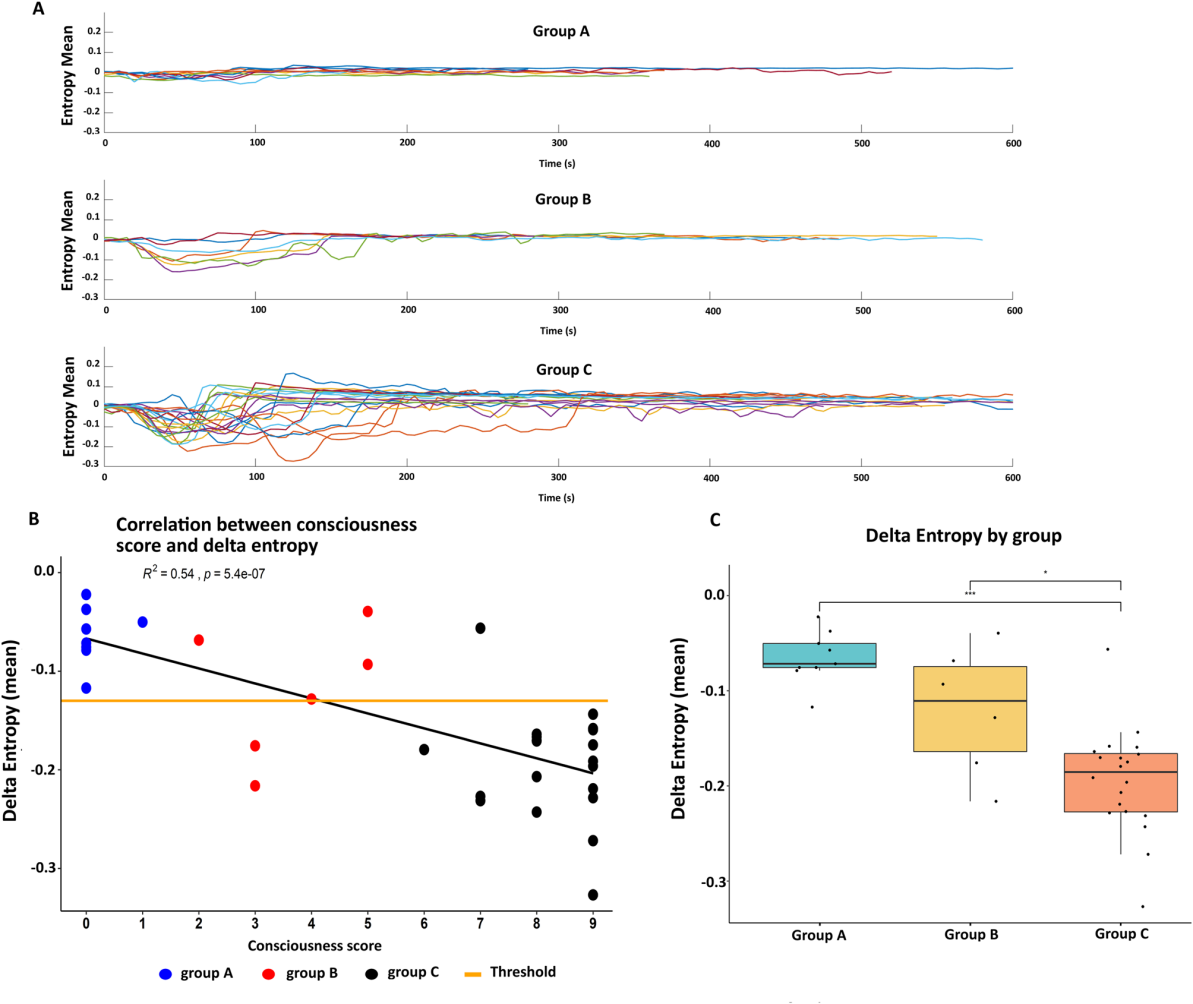
Visualization of the entropy changes by consciousness groups (Group A, B, C). (**A**) Mean entropy values averaged from all channels for all the seizures in each group. (**B**) Correlation between consciousness scores and mean delta entropy value with the threshold determined by ROC curve to differentiate Group C and Group A (threshold in orange at − 0.135) (**C**) Distribution of the delta entropy values according to each group. * p < 0.05 *** p < 0.001.

As shown in Fig. 2A, three different patterns of ictal entropy dynamics were obtained for each group. A ΔE close to zero was seen in group A, while a high negative ΔE (corresponding to the lowest values of PE) was observed for Group C. Group B seizures disclosed intermediate ΔE values. There was a significant correlation between the mean ΔE values and the CSS scores (Fig. 2B). The difference in the mean ΔE values was statistically significant (p < 0.001) between the groups A and C (Fig. 2C) and to a lesser extent between the groups C and B (p < 0.05). No correlation was found between the ∆_time_ and the consciousness score.

### Thresholded ΔE values to discriminate between the groups A and C

By using ROC method, we obtained two thresholds of ΔE that can distinguish the group, one at − 0.13 and the other at − 0.14 (Fig. 1S in Supplemental Material). Both these thresholds allowed perfect distinction between the group A and group C on our dataset (Fig. 2B). We thereby used the averaged threshold value of ΔE at − 0.135 for our analysis. Values in Group B were varying around the threshold (that was optimized for classification of C versus A) and showed larger distribution suggesting that this group is more heterogeneous.

### Entropy alterations in temporal and extra-temporal regions within groups A and C

**Figure 3:**
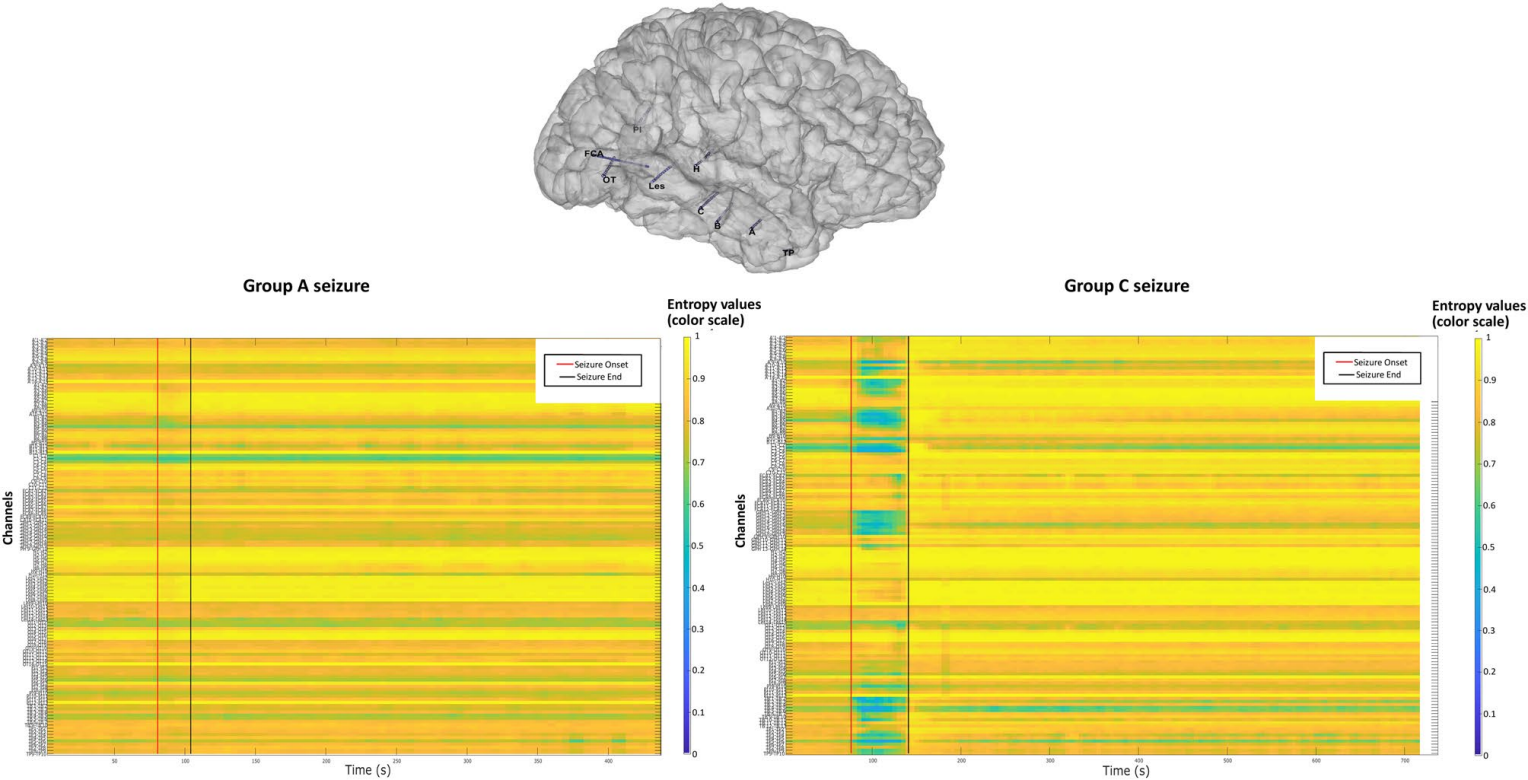
Stereotactic-EEG electrodes implantation of one subject having one seizure in Group A and one seizure in Group C. The red vertical line corresponds to seizure onset and the black vertical line, to seizure offset as determined visually by the scorer based on the SEEG signal.

Figure 3 shows an illustrative example of a patient with two different seizures, one with no alteration of consciousness (group A) and another with severe alteration of consciousness (group C): Remarkably, these seizures disclose clearly different patterns of entropy changes, with more regions involved when alteration of consciousness is observed.

**Figure 4:**
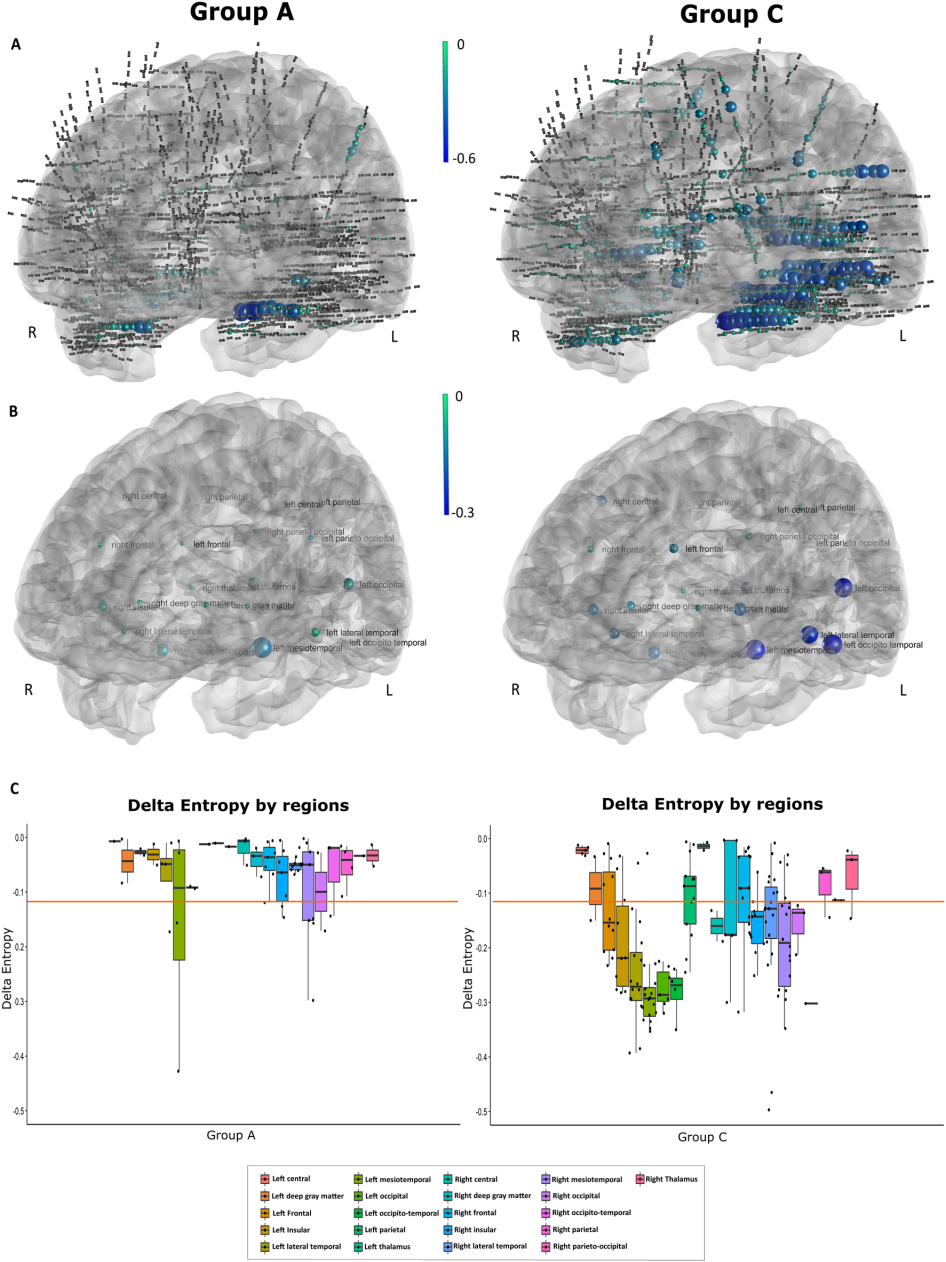
Visualization of the delta entropy values inside the brain. (**A**) Stereotactic-EEG electrodes of all subjects are projected into MNI template using GARDEL^22^ software allowing a 3D vision of the contacts. Then delta entropy value by contact for Group A and C are visualized, the size and the color being proportional to the values. (**B**) Visualization of delta entropy by anatomical regions for Group A and C. (**C**) Distribution of delta entropy values by anatomical regions with the threshold at − 0.135 (in orange).

Since we observed a clear relationship between global ΔE and awareness level, we then investigated the variations of ΔE across different brain regions defined as explained above. We visualized the ΔE values in the patient’s anatomy to assess which regions are more involved with regards to the alteration of awareness, by comparing Group A with Group C seizures. Figure 4A illustrates all electrodes of all patients of each respective group represented on a brain 3D mesh, with the mean ΔE values of each bipolar contact represented as spheres according to a color scale. Compared to the group A, group C seizures were characterized by a greater number of involved contacts in both hemispheres. The ΔE values for each anatomical region are indicated in Fig. 4B and C. Numerous regions of both hemispheres disclosed changes in delta entropy and thus showed high negative delta entropy values in Group C (left frontal, left insula,left lateral temporal, left mesiotemporal, left occipital, left occipito-temporal, right central, right insula, right lateral temporal, right mesiotemporal and right occipital), while few were involved in Group A.

## Discussion

This is the first study to our knowledge that evaluates permutation entropy in relationship with alteration of awareness in focal seizures. We chose to study a relatively homogeneous group of patients with temporal lobe seizures. Our results reveal a positive correlation between the delta entropy (ΔE) and the consciousness score. Furthermore, we were able to define a threshold of the entropy changes, measured with ΔE, allowing to discriminate between the seizures of group A (no alteration of awareness) and group C (severe alteration of awareness) Another important finding is that in patients with loss of awareness unlike in those with preserved awareness, the demonstrated changes in ΔE values showed more extended spatial distribution.

As far as various disorders of consciousness (DoC, e.g., syncope, coma, vegetative states, sleep disorders, encephalopathy and seizures) are concerned, it is the degree of impairment within the “consciousness system” that defines the extent of alteration of consciousness^23^. Numerous studies have been conducted to identify the components of consciousness in patients with DoC and, most importantly, to identify physiological markers in the clinical setting and thereby to better define the patient’s prognosis.

With respect to the structural imaging, findings demonstrated to be specific for patients with permanent DoC (Vegetative state, VS, and minimally conscious state, MCS) as compared to healthy controls, include diffuse abnormalities in the cortical, subcortical, and white matter volume and connectivity^24^.

The use of entropy allows a successful evaluation of the "quantity of information" or the "complexity" of a system. Several theories predict that the complexity of information integration^25^ or distributed processing^26^ is elevated during conscious states. Permutation entropy assesses the regularity of the probabilistic distributions of temporal patterns in the signal^21^.

Permutation entropy has been studied by different teams in various DoC. Thul^27^ found reduced permutation entropy on scalp EEG in patients with minimally conscious state and in the unresponsive wakefulness syndrome (UWS) compared to healthy controls. Furthermore, this reduction was most pronounced in the UWS group.

Another method developed by King^28^ based on weighted symbolic mutual information (wSMI) allowed discrimination between VS and MCS. Sitt and colleagues^29^ used different metrics obtained from scalp EEG to discriminate between different states of consciousness (low frequency power, EEG complexity and information exchange). They found that permutation entropy-based measures were particularly efficient in the theta frequency range to discriminate VS from the other groups. PE has been widely used as a tool to track the qualitative effect of anesthetics throughout the different stages (awake, to slightly and deeply sedated)^30,31^ becoming a means of evaluating the depth of anesthesia monitoring^32–34^.

These studies confirmed that access to consciousness requires sufficient information content resulting in high signal complexity whereas altered states of consciousness are associated with low signal complexity.

Li^31^ found that permutation entropy can be used to predict absence seizures using genetic absence epilepsy rats from Strasbourg (GAERS) model, and to demonstrate the presence of a pre-ictal state in absence seizures: a dynamic progressive decrease in PE was observed before seizure onset. Furthermore, Ferlazzo^35^ showed higher permutation entropy in fronto-temporal areas and lower entropy values in posterior areas, during interictal and ictal periods, in patients with absence seizures in comparison to healthy controls.

The originality of our study is the use of permutation entropy in the context of alteration of consciousness occurring in patients with TLE, recorded with intracerebral electrodes (SEEG). In contrast with other disorders of consciousness, this state is transient and must be captured during the seizure periods. Taken as a whole, we observed a decrease of PE values at the seizure onset and throughout the seizure spread in the brain. This decrease was more pronounced in patients with an alteration of awareness. We estimated the decrease from baseline by a metric called “delta entropy” (∆E) and we found a positive correlation between the ∆E value and the consciousness score: Group A seizures were characterized by a ∆E value close to zero and Group C seizures with a high negative ∆E. This means that during a seizure with no alteration of consciousness the variation in permutation entropy between the baseline and the minimal entropy value obtained is smaller in comparison to a seizure with severe alteration of consciousness. These results suggest that seizures with maximal alteration of consciousness also have maximal loss of signal complexity. We can thus observe a spectrum of decreased delta entropy as we go from group A to group C, which also applies to the extent of the regions involved as we move along these groups. Indeed, we noted an involvement of numerous brain regions in Group C seizures, while in Group A seizures, mostly the temporal lobe structures (left and right) were involved.

Thus, it appears that the loss of complexity observed in group A seizures is limited to temporal structures. The associative cortical regions keep their level of complexity, probably compatible with information exchange and conscious processing.

In group C seizures, the loss of signal complexity was more diffuse, extending bilaterally as well as to the associative cortices. In term of pathophysiology, these results suggest that the loss of signal complexity becomes incompatible with conscious processing. This is in agreement with different theories of consciousness based on information theory (IT)^36^, re-entrant processes (RP)^37^ and global workspace theory (GW)^13^. Indeed, the relationship with the level of complexity alteration at the local level is in agreement with the IT or RP, while the extension to extra-temporal cortices is in agreement with an interruption of the information flow in the GW. Noteworthy, no clear pattern was seen in group B seizures, where the delta entropy values were more heterogeneous. This can be explained by the fact that the extent of alteration of consciousness in this group is more diverse, since the consciousness scores vary between 2 and 5 and thus these seizures can behave either like group A or group C seizures.

Furthermore, no positive correlation was found between the delta time (∆_time)_ and the consciousness score in any group. This could be compatible with the observation that the duration of a seizure does not necessarily determine the extent of alteration of consciousness: an isolated typical epileptic aura can last few minutes and usually does not impair the components of consciousness.

Finally, despite some limitations mentioned below, our results suggest that the delta entropy marker can be used as a potential predictor tool for assessing the extent of alteration of consciousness^38^.

## Limitations

A limitation to our study is the small sample size: in fact, more data is needed to properly apply the threshold entropy marker and its usage as a predicting tool in terms of the extent of alteration of consciousness. Another limitation is the lack of sufficient electrode sampling of some regions: in the setting of temporal lobe epilepsy, considered as the main hypothesis during the pre-surgical evaluation, some cases did not have enough sampling of the extratemporal, particularly in the parietal regions. Additionally, not all patients had subcortical contacts in the thalamus. Thus, the extent of involvement of the thalamus and parietal regions remains not clearly defined by the current data.

Furthermore, the calculation of the threshold was determined on the same dataset used for the analysis; this method needs to be applied on another dataset to confirm the threshold value.

## Conclusion

Permutation entropy is a promising tool to discriminate between the different levels of consciousness alteration in seizures, and mainly between group A and group C by using a threshold value of delta entropy. Both the value of entropy changes and the spatial extent probably account for the consciousness alterations. This metric is relatively easy to use, especially when compared to connectivity methods (i.e. h^2^ method^14–16^). It could then be implemented to estimate the therapeutic impact of certain neuromodulation techniques on improving consciousness during seizures, as observed with VNS^39–42^ or DBS^43,44^. Future studies are needed to study permutation entropy and alteration of consciousness in the setting of extra-temporal epilepsies.

## Materials and methods

### Patients’ selection

Patients were retrospectively selected from our database of focal pharmaco-resistant epilepsy cases undergoing pre-surgical evaluation within the Epilepsy Unit at the Timone Hospital in Marseille using SEEG recording between October 2015 and December 2018. A systematic patient examination and interview has been conducted by nurses or physicians during seizures to assess the level of interaction as well as language impairment^45,46^. The study was approved by the institutional review board (Assistance Publique Hôpitaux de Marseille APHM 2021–179) and informed consent was obtained from each subject at the time of SEEG recording. All experiments were performed in accordance with relevant guidelines and regulations.

### SEEG recordings

Recordings were performed using multi-contact depth electrodes (10–15 contacts, length: 2 mm, diameter: 0.8 mm, 1.5 mm apart) placed intracerebrally according to Talairach’s stereotactic method^47^. Signals were then recorded on a 128 or 256 channel Deltamed TM system and amplified at 256, 512 or 1024 Hz and recorded with no digital filter. Two analog filters were used at the time of recording: a high-pass filter (cut-off frequency equal to 0.16 Hz at -3 dB) and an anti-aliasing low-pass filter (cut-off frequency equal to 97 Hz at 256 Hz, or 170 Hz at 512 Hz or 340 Hz at 1024 Hz).

### Estimation of awareness impairment using the consciousness seizure scale (CSS)

We analyzed the alteration of awareness using the Consciousness Seizure Scale, CSS^14^. The scoring was done by two neurologists (NEY and FB) and the mean of the two scores was taken. The CSS relies on an eight-criterion scale taking into account the different components of consciousness: (1) unresponsiveness, (2) visual attention, (3) interaction, (4) consciousness of the seizure, (5) adapted behavior, (6) post-ictal amnesia and (7) interictal amnesia. Finally, the (8) “global appreciation criterion” based on the physician global assessment and scored between 0 and 2. The timing of the maximal alteration of consciousness (as determined by the scorer) was set as Tmax.

According to our previous studies^14,15,45^, the seizures were classified into Group A (score 0 to 1), Group B (score 2 to 5) and Group C (score 6 to 9) with Group A corresponding to preserved awareness, Group B to intermediate alteration of awareness and Group C to profound alteration of awareness.

### Permutation entropy method

In this study, we chose entropy to characterize the level of disorganization of the signals and to define the timing of maximal LOC as well as spatial extent of alteration of consciousness. The specific entropy method chosen was the “Permutation Entropy”, PE^21^, which takes into account the complexity of the electrophysiological signals. PE is based on a measure of Shannon entropy of the relationships between neighbor values of the time series.

The method is rendered more robust than standard information-based method by calculating the entropy for n “embedding dimensions” in the range of 3–7, (i.e., different number of neighbors or samples). We used a fixed delay τ which indicates the distance between neighbors (τ = 1 for consecutive samples).

$$H(n)= -\sum p\left(\pi \right)\mathrm{log}p\left(\pi \right)$$

With π describing the dynamics between the neighbor values (e.g.,${x}_{t}< {x}_{t+1}$), $p\left(\pi \right)$ probability of this order and n embedding dimensions. H (n) gives a value between 0 and 1, 0 meaning that the signal is perfectly organized (for example in the case of a pure sine wave) and 1 completely disorganized (completely random noise). The algorithm used to apply permutation entropy on our data is the one developed by Unakafova^48^ due to its algorithmic performance. The efficiency of this method stems from the use of pre-filled tables to describe the order relation between neighbor values.

To normalize and compare the entropy time course across patients and seizures, we selected seizures with a minimum baseline of 30 s before seizure onset. Before applying permutation entropy on a given seizure signal, we performed careful selection of channels by keeping those in the grey matter and removing the white matter channels. A bipolar montage was used (i.e., difference of consecutive channels). Channels with artefact or high level of noise were removed. Permutation entropy was computed on each channel, on a sliding window of 10 s with an overlap of 5 s, an order of 3 and a delay of 1 sample. We studied entropy at two different scales: on a global level by averaging all SEEG contacts, and on a regional level by using anatomical regions (see Supplementary material for more details). For these two different scales, each entropy signal was normalized by subtracting the mean of the baseline channel by channel. To measure changes in entropy, we used two different variables named “delta entropy” (ΔE), and “delta time” (∆_time_) (Fig. 1). “Delta entropy” is the difference between the entropy value at the baseline and the minimum entropy reached during the seizure, “Delta time” is the time between the end of baseline (corresponding to the beginning of the seizure) and the time at which the minimum entropy occurs. Figure 1 shows these two variables, the signal markers that were set by the scorer corresponding to the beginning and end of the seizure (T0 and Tend) and the time of maximal alteration of consciousness (for groups B and C) as defined by the CSS.

For the global level analysis, we calculated the mean entropy by using all channels of the montage, allowing to test for a global difference across the groups and to determine a threshold separating the seizures of Group A and Group C. Using the classification, A versus C, we computed the false positive rate, the true positive rate and the F1 score of the different threshold values (-0.3: -0.01:0) of delta entropy. We thus obtained a ROC curve leading to an optimal threshold (supplemental fig. S1). All the seizures with a delta entropy value inferior to the threshold were labelled as Group C (Fig. 3).

CT-scan with electrodes/preoperative MRI data co-registration was performed using the open-source software GARDEL^22^ and projected into the MNI template for 3D representation shown in Fig. 4. For the regional level analysis, each bipolar SEEG contact was assigned to a brain region by using an automated parcellation according to the VEP atlas^49,50^ with accuracy visually checked by a neurologist. For the purpose of this study, the following anatomical regions (left and right hemispheric) were defined by regrouping several VEP atlas regions: mesio-temporal, lateral temporal, central, frontal, parietal, occipital, insular, occipito-temporal, parieto-occipital, deep gray matter, thalamus (see Supplementary material for details). Only the regions sampled in at least two seizures were kept for the analysis. For each anatomical region and each subject, the delta entropy values (ΔE) from all contributing channels were averaged, resulting in one value per region. By assessing the ΔE per anatomical region for all the seizures of each respective group (A, B and C), we estimated which brain regions are more involved during the change in awareness. However, due to the limitations inherent to the SEEG method, the number of contacts per region differed between the patients and the number of contributing subjects differed between the regions, resulting in a lack of statistical power of such analysis (Fig. 1, Supplemental material).

### Statistical analysis

We computed the linear correlation between the consciousness score and delta entropy using Pearson method. Then a linear mixed-effect model (LMM) using the “lme4” package (version 1.1–26^51^, of the statistical software R version 4.0.3 (R Core Team, 2020) was used to study the effect of the group (A, b or C) on the ΔE value, with subject as random effect.

Restricted Maximum Likelihood (REML) was used to fit the LMM and Satterthwaite’s method was used to estimate the degrees of freedom. Post-hoc pairwise comparisons were performed with the Least Square Means (LSM) method. Importantly, the use of a mixed model allows to take into account variability across patients (Table 2, Supplemental material).

Concerning the different anatomical regions, due to the above-mentioned limitations, it was impossible to perform a statistically powerful analysis of the ΔE per region to see the effect of the group, neither a statistical comparison between the regions and the seizure groups.

## Supplementary Information

Supplementary Information.

## Data Availability

The datasets used and/or analysed during the current study available from the corresponding author on reasonable request.
