# Supplementary material for: Consciousness alteration in focal epilepsy is related to loss of signal complexity and information processing
Source: Sci Rep. 2022 Dec 24;12:22276. doi: 10.1038/s41598-022-25861-4 (PMC9789957; doi:10.1038/s41598-022-25861-4)
Supplement: Supplementary file 1 — Supplementary Information. [file 41598_2022_25861_MOESM1_ESM.docx]

**
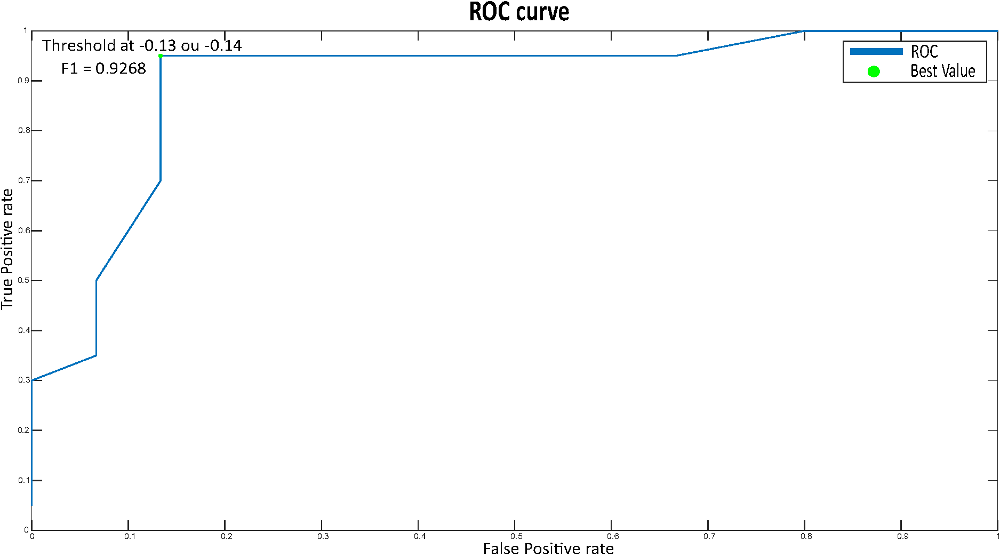
**

**Figure 1S:** Receiver Operating Characteristic curve. This curve is the representation of the classification with different thresholds to determine the delta entropy threshold associated to Group C (with respect to Group A). The best threshold obtained is the one that gives the lower false positive rate with the higher true positive rates

**
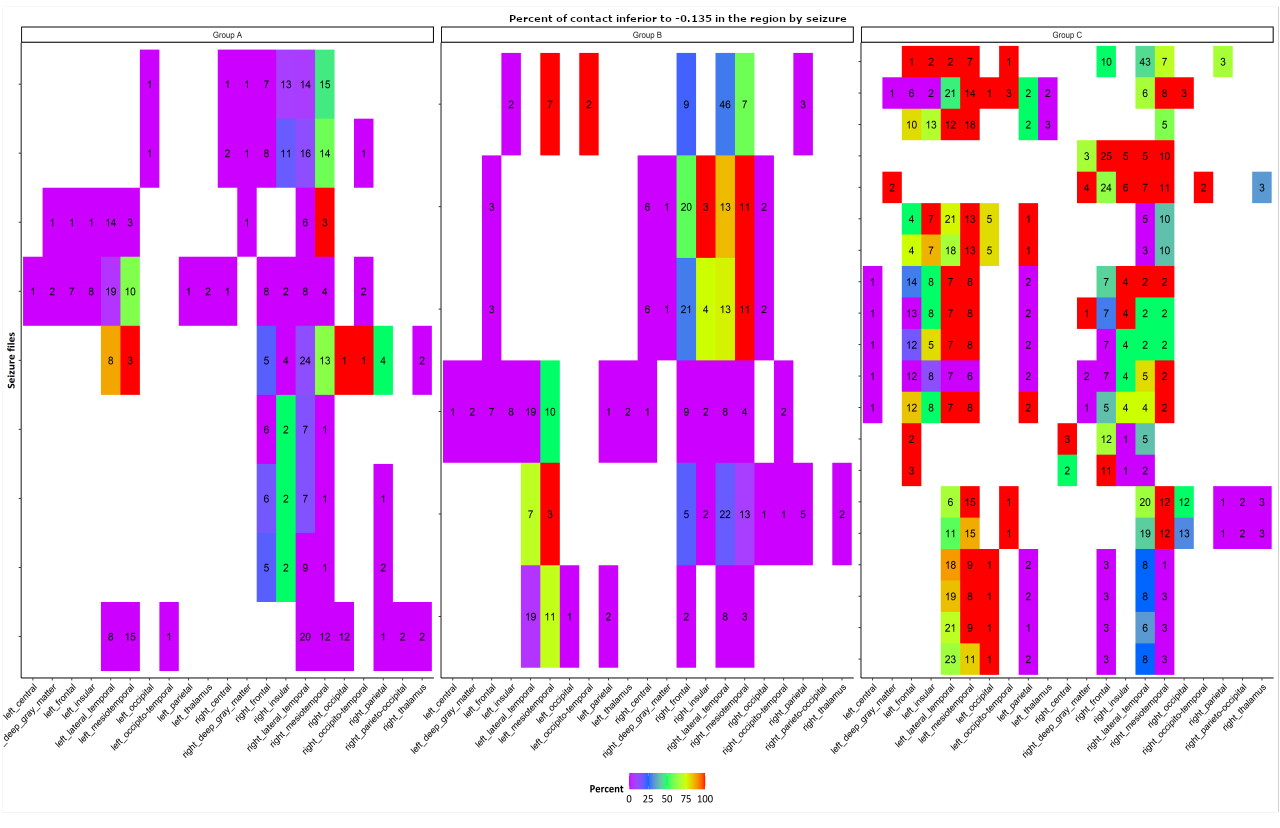
**

**Figure 2S**: This figure represents the number of contacts present in the region for group A, group B and group C. The number of contacts is written in black. Moreover, the color represents the percentage of contacts with delta entropy below the specific threshold (-0.135) with red color corresponding to all the contacts above this threshold and purple color to none of them above the threshold. By comparing the different groups, the number of contacts in the anatomical regions is heterogenous, some regions having a low number of contacts. The different colors give the percentage of contacts with an ΔE below the previously obtained threshold

**Table 1: Table of the selected anatomical subregions**

| (Left/ right) mesiotemporal | (Left/right) Amygdala; Hippocampus; Rhinal-cortex; Para hippocampal cortex; Collateral-sulcus. |
| --- | --- |
| (Left/ right) latero-temporal | (Left/right) Gyrus-of-Heschl; ITS-anterior; ITS-posterior; STS-anterior; STS-posterior; T1-lateral anterior; T1-lateral posterior; T1-planum polare; T1-planum temporale; T2-anterior; T2-posterior; T3-anterior; T3-posterior; Temporal-pole; Fusiform-gyrus. |
| (Left/ right) central | (Left/right) Central operculum; Precentral gyrus head face; Precentral sulcus inferior part; Precentral sulcus superior part; Central sulcus head face; Precentral gyrus upper limb. |
| (Left/right) frontal | (Left/right) Anterior cingulate cortex; F1 lateral premotor; F1 mesial prefrontal; F2 caudal; F2 rostral; F3 Pars triangularis; F3 pars opercularis; Gyrus rectus; Inferior frontal sulcus; Middle frontal sulcus; Orbitofrontal cortex; SFS caudal; SFS rostral; Subcallosal area; Middle cingulate cortex anterior part; Middle frontal sulcus; Pre-SMA. |
| (Left/ right) parietal | (Left/ right) Angular gyrus; Parietal operculum; Posterior cingulate cortex dorsal; Precuneus; Supramarginal anterior; Supramarginal posterior; Postcentral sulcus. |
| (Left/ right) occipital | (Left/ right) Anterior occipital sulcus and pre-occipital notch; Fusiform gyrus; Calcarine sulcus; Lingual gyrus; Lingual sulcus; O1; O2. |
| (Left/ right) insular | Insula gyri brevi ; Insula gyri longi. |
| (Left/ right) occipito- temporal | Occipito-temporal sulcus. |
| (Left/ right) parieto-occipital | Parieto-occipital-sulcus. |
| (Left/right) deep gray matter | (Left/right) Caudate; Putamen; Ventral DC. |
| (Left/right) thalamus | (Left/right) Thalamus Proper. |

|  | **Δ Entropy** | | | |
| --- | --- | --- | --- | --- |
| *Predictors* | *Estimates* | *CI* | *p* | *df* |
| Intercept | 0.07 | 0.03 – 0.12 | **0.001** | 23.81 |
| Group B | 0.03 | -0.02 – 0.09 | 0.219 | 31.03 |
| Group C | 0.13 | 0.08 – 0.18 | **<0.001** | 29.22 |
| **Random Effects** | | | | |
| σ^2^_subject_ | 0.0014 | | | |
| σ^2^_residuals_ | 0.0015 | | | |
| ICC | 0.48 | | | |
| N _subject_ | 15 | | | |
| Observations | 35 | | | |
| Marginal R^2^ / Conditional R^2^ | 0.560 / 0.772 | | | |

**Table 2: Description of the statistical analysis method**
